# Deep structured populations of geographically isolated nipa (*Nypa fruticans* Wurmb.) in the Indo-West Pacific revealed using microsatellite markers

**DOI:** 10.3389/fpls.2022.1038998

**Published:** 2022-10-25

**Authors:** Junaldo A. Mantiquilla, Meng-Shin Shiao, Hsueh-Yu Lu, Kitichate Sridith, Siti Nordahliawate M. Sidique, Wasantha Kumara Liyanage, Ya-Ling Chu, Huie-Chuan Shih, Yu-Chung Chiang

**Affiliations:** ^1^ Department of Biological Sciences, National Sun Yat-sen University, Kaohsiung City, Taiwan; ^2^ Department of Biological Sciences and Environmental Studies, University of the Philippines Mindanao, Davao City, Philippines; ^3^ Research Center, Faculty of Medicine Ramathibodi Hospital, Mahidol University, Bangkok, Thailand; ^4^ Department of Biology, Prince Songkla University, Hat Yai, Thailand; ^5^ Laboratory for Pest, Disease and Microbial Biotechnology (LAPDiM), School of Food Science and Technology, Universiti Malaysia Terengganu, Kuala Terengganu, Malaysia; ^6^ Department of Agricultural Biology, Faculty of Agriculture, University of Ruhuna, Kamburupitiya, Sri Lanka; ^7^ Department of Nursing, Meiho University, Pingtung, Taiwan; ^8^ Department of Biomedical Science and Environment Biology, Kaohsiung Medical University, Kaohsiung, Taiwan

**Keywords:** nipa, Indo-West Pacific, microsatellites, genetic diversity, population structure, migration

## Abstract

Nipa (*Nypa fruticans* Wurmb.) is an important mangrove palm species, but it is understudied due to lack of information on genetic patterns within its distribution range. In this study, we identified 18 informative microsatellite markers to assess genetic variations among local populations in the Indo-West Pacific (IWP). Results showed population stratification based on high genetic differentiation (F_ST_ = 0.22131) with the Mantel test indicating significance to isolation-by-distance. We found a pronounced differentiation between the west populations in Sri Lanka and east populations in Southeast Asia. The east populations around the South China Sea were more genetically similar than those along the Malacca Strait and Java Sea. These genetic clines were shaped by ocean circulations and seasonal monsoon reversals as plausible factors. The Malacca Strait was confirmed as both a genetic and a geographic barrier rather than a corridor according to the Monmonier plot. Simulations of directional migration indicated a statistically strong contemporary genetic connectivity from west to east where Sri Lankan immigrants were detected as far as central Philippines *via* long-distance dispersal. This is the first report on the recent migration patterns of nipa using microsatellites. Assignment of first-generation (F_0_) immigrants suggested Mainland Southeast Asia as a melting pot due to the admixture associated with excess of homozygosity. The western populations were recent expansions that emerged in rapid succession based on a phylogram as supported by footprints of genetic drift based on bottleneck tests.

## Introduction

Earth is a plant-oriented planet and underutilized plants are of special importance. Underutilized plants are adding value to earth’s diversity that is fundamental to all life. They have multiple uses such as food and beverages, landscape arranging, and decorative household items, among others, with significant economic contribution (Dogan et al., 2014; Hasanbegovic et al., 2021; Saran et al., 2021). Among the palms, nipa serves as a source of construction materials (primarily thatch), dessert, alcoholic beverage, and bioethanol, and holds great economic potential, but it remains understudied (Rasco, 2011).

The nipa palm (*Nypa fruticans* Wurmb.) is the only species in the genus *Nypa* and in the subfamily Nypoideae, which indicates the distinct morphological characteristics from other members of the family Arecaceae (Uhl, 1972; Dransfield et al., 2008). It has an underground rhizomatous stem that grows horizontally and exhibits dichotomous branching, which can produce several ramets in a population. Once the branch is cut off from the mother palm, it becomes a new plant by clonal propagation (Rasco, 2011). However, nipa reproduces primarily by outcrossing, because the anthesis of protogynous female flowers occurs ahead of the males (Henderson, 1986; Mantiquilla et al., 2013). This natural cross-pollination strategy is entomophilous, which results in a concomitant high degree of self-incompatibility (Mantiquilla et al., 2016).

Along with other true mangroves, nipa constitutes the wetlands as influenced by tides that distribute mostly in Southeast Asia. It is particularly found in the estuaries trapping suspended sediments, where it stands to serve as a transition belt between mangrove and freshwater swamps (Fong, 1992). The current distribution of nipa is confined within the tropical Indo-West Pacific Region that ranges from Sri Lanka through Southeast Asia to northern Australia and the western Pacific islands (Hossain and Islam, 2015). Nakanishi (1987) reported that nipa also occurs irregularly in Iriomote Island in Ryukyu, Japan, which is the northern limit of nipa distribution. However, the individual plants were found sporadically on the island and were not able to establish the population due to lower average temperatures.

The fruit of nipa is a woody drupe that forms a cluster compressed into a fruit head termed as “infructescence”. The mature infructescence disintegrates by incipient vivipary; i.e., the fruits initiate to germinate by a protruding plumule followed by pushing them to release from the head and dispersed by tide floating (Das and Ghose, 2003; Joshi et al., 2006). This buoyancy is secured by the transformation of the outer mesocarp of the drupe into aerenchyma after the fruit attains its final dimensions, allowing hydrochory dispersal (Bobrov et al., 2012).

The germinated seed may drift on water in motion by either the flowing river or surface currents caused by wind in oceanic circulations, which may result in long-distance dispersal. Nakanishi (1987) recovered 28 species of drift disseminules, including nipa, on the mainland coasts of Japan. These driftages, mostly of palaeotropical types, were washed up from the Japan Sea along the entire west coast, but limited on the east coast facing the Pacific Ocean (PO).

pt?>Different genetic markers have been used to estimate the levels of genetic diversity and the degree of differentiation between and among nipa populations. These genetic markers include random amplified polymorphic DNA (RAPD), amplified fragment length polymorphism (AFLP), inter simple sequence repeats (ISSR), and simple sequence repeats (SSR). However, different conclusions have been drawn in different geographic regions using different markers. Several studies reported no or low levels of genetic diversity within and between populations in different areas including West Bengal, India (Bandyopadhyay et al., 2005), Iriomote Island, Japan (Setoguchi et al., 1999; Sugai et al., 2015), and Hainan, China (He et al., 2015). By including samples from a larger geographic region, i.e., China, Vietnam, and Thailand, Jian et al. (2010) reported polymorphisms in 2 SSRs among a total of 17 SSRs. In contrast, two studies show relatively higher levels of genetic variation by examining more genetic markers and more populations around Southern Luzon, Philippines (Lapitan and Nicolas, 2015) and Peninsular Malaysia (Tsuji et al., 2016). The authors identified polymorphisms in 25 SSRs among a total of 31 SSRs using 163 collected accessions, and in 57 AFLP loci among a total of 242 AFLP loci based on 27 populations examined, respectively. The inconclusive evidence of genetic diversity and differentiations may be due to less informative markers and limited sampling sites in the distribution range, or it truly demonstrated the uneven population diversities in different geographic regions of nipa.

As the species disperses widely in IWP and the genetic variations of its populations are still inconclusive, we aim to elucidate the population differentiation of nipa by extensive collection of samples in IWP and apply SSR markers to answer the question. More specifically, we will measure the genetic diversity of the local populations, determine the population structure and the associated genetic clustering in IWP, and evaluate the biological processes shaping the phylogeography of nipa for future conservation strategies.

## Materials and methods

### Sample collection and DNA isolation

A total of 445 samples were collected from different geographic regions in IWP. Sample collection began in May 2018 to October 2019 that ranged from the Philippine Archipelago, mainland Southeast Asia (Vietnam, Thailand, Malaysia, and Singapore), Indonesia, and Sri Lanka. The sampling sites in the region are enumerated in **Table 1**.

**Table 1:** The different sampling sites of nipa populations and their corresponding sampling sizes in the Indo-West Pacific.

Population code	Sampling sites	Sample size
**A. Philippine Archipelago (PH)**		
1. Mindanao South (PHMS)	Carmen/Tagum, Davao del Norte	15
2. Mindanao East (PHME)	Bislig, Surigao del Sur	18
3. Mindanao North 1 (PHMN1)	Placer, Surigao del Norte	6
4. Mindanao North 2 (PHMN2)	Cabadbaran/Magallanes, Agusan del Norte	12
5. Central (PHCE)	Baclayon/Cortes/Loboc, Bohol	19
6. Luzon East (PHLE)	Cagsiay/Lual, Mauban, Quezon	15
7. Luzon West (PHLW)	Labrador/Lingayen, Pangasinan	18
8. Palawan West (PHPW)	Nagtabon Beach, Palawan	6
9. Palawan East (PHPE)	Sta. Monica/Sicsican, Palawan	9
**B. Mainland Southeast Asia**		
**1. Mainland Indo-China (ML)**		
a. Vietnam 1 (MLVN1)	Ho Chi Minh	15
b. Vietnam 2 (MLVN2)	Ba Lai River/An Khanh, Ben Tre	21
c. Thailand 1 (MLTH1)	Samut Prakan 1/2	15
d. Thailand 2 (MLTH2)	Samut Songkhram/Tambon Mae Klong/ PK Soi 3	16
e. Thailand 3 (MLTH3)	Chachoengsao	16
**2. Malay Peninsula (MP)**		
a. Northern Coast 1 (MPNC1)	Pa Khat/Pak Ro/Cha Lae/Tambon Cha Lae/Bang Khiat	20
b. Northern Coast 2 (MPNC2)	Bang Riang/Tambon Bang Riang	15
c. Northern Coast 3 (MPNC3)	Tambon Ko Yo/Songkhla Lake/Ko Yo	16
d. East Coast 1 (MPEC1)	Mangkuk/Jerteh/Permaisuri/Kuala Terengganu	26
e. East Coast 2 (MPEC2)	Marang. Terengganu	15
f. East Coast 3 (MPEC3)	Kemaman, Terengganu	15
g. East Coast 4 (MPEC4)	Johor, Mersing	15
h. East Coast 5 (MPEC5)	Pekan, Pahang	15
i. Southern Coast (MPSC)	Singapore	20
j. Southwest (MPSW)	Negeri Sembilan	21
**C. Indonesia (INNJ)**	North Jakarta	7
**D. Sri Lanka (SL)**		
1. Southwest 1 (SLSW1)	Balapitiya	12
2. Southwest 2 (SLSW2)	Dondra/Talalla	12
3. Southwest 3 (SLSW3)	Matara	8
4. Southwest 4 (SLSW4)	Hapugala	8
5. West Coast 1 (SLWC1)	Negombo	6
6. West Coast 2 (SLWC2)	Nainamadama/Kammala/Wennapuwa	11
7. West Coast 3 (SLWC3)	Puttalam	2
**Total**		**445**

The leaflet samples were kept refrigerated in tea bags with silica gel for desiccation prior to DNA isolation. The DNA was then extracted using a kit (RBC Real Genomics™ Box YGP 100) replicated three times followed by quality and quantity assessment: DNA quality and concentration were checked with the 1.5% agarose gel electrophoresis and Nano-300 Microspectrophotometer (Allsheng, Hangzhou, China), respectively. Samples with DNA absorbance ratio (A260/A280) larger than 1.8 were used for further analyses.

### Microsatellite analysis

A total of 18 SSR loci identified as informative in the previous study was based on the whole genome sequencing of nipa by DNA amplification and polymorphic screening (Mantiquilla et al., 2021) intended for the 445 samples. Polymerase chain reactions (PCRs) were performed with a total of 20-µl volume containing 10.6 µl of double-distilled water, 2 µl of 10× buffer, 2 µl of dNTP, 2 µl of each of the forward and reverse primers, 0.4 µl of BSA, 0.5 µl of sample DNA, and 0.5 µl of *Taq* polymerase. Thermal cycles were performed in a Multigene Optimax Thermal Cycler (Applied Biosystems, CA, USA): initial denaturation at 94°C for 2 min, which was followed by 35 cycles of denaturation at 94°C for 45 s, temperature gradient between 50 and 60°C at 45 s, and extension at 72°C for 50 s. The final extension was set at 72°C for 7 min. The PCR products were then checked with 1.5% agarose gel electrophoresis prior to loading appropriate amount to polyacrylamide gel to read polymorphism.

Polymorphisms of 18 SSR loci were finally assessed by 10% polyacrylamide gel electrophoresis of PCR products mentioned above. The banding patterns were visualized by staining with ethidium bromide and by imaging with a digital camera. All markers were scored as co-dominant data based on the amplicon size using Quantity One ^®^ software (Bio-Rad) to create a data matrix manually (**Table S1**).

### Population genetic analyses

All data analyses and visualization were conducted using R Stats Package (R Core Team, 2021) unless indicated otherwise. The genetic diversity and structure of nipa populations in IWP were analyzed for the following parameters (McDonald, 2008):

- Variations at the population level were estimated by the number of alleles per locus, heterozygosity (observed and expected), population’s fixation index (F), and % polymorphic loci (P) in a population. Also, the analysis of multilocus genotype (MLG) diversity detects linkage disequilibrium (LD), a test for the departure from panmictic population by the standardized index of association, rbarD (r̄_d_), which accounts for the number of loci using *poppr* in R. Moreover, the identification of candidate loci under natural selection was determined using Bayescan based on reversible-jump Markov chain Monte Carlo (MCMC).
- Variations at different levels among populations were assessed by the analysis of molecular variance (AMOVA), Wright’s fixation indices (F_IS_, F_ST_, and F_IT_), and principal coordinates analysis (PCoA). Correlation between genetic distance and geographic distance was determined using Mantel tests (Diniz-Filho et al., 2013) for isolation by distance.
- Population structure based on spatial patterns of genetic connectivity was analyzed by the spatial principal component analysis (sPCA) using *adegenet* in R. The genetic boundaries among georeferenced populations were detected by Monmonier’s algorithm. It finds the boundaries of maximum differences between continuous polygon of Voronoi tessellation to identify genetic barriers.
- Population structure with assignment tests was evaluated by MCMC for genetic clusters based on the output summary of STRUCTURE HARVESTER implemented according to the Evanno method using STRUCTURE and *Geneland* (for georeferenced genotypes) that run under a Bayesian model, and the discriminant analysis of principal components (DAPC), using *adegenet* in R.
- Directional migration patterns (east to west and *vice versa*) were tested according to relative migration network presented here based on *Nm* as determined by *diveRsity*, while the phylogenetic tree was constructed by a phylogram using the neighbor-joining method according to Nei’s distance by *adegenet*; both are implemented in R.

## Results

### Structured populations of nipa in IWP

Previously, we reported that 18 out of 75 SSR loci were identified as highly polymorphic from 37 random samples (Mantiquilla et al., 2021). This initial screening indicated that the 18 SSRs are informative for genetic diversity analysis. Therefore, we expanded the samples to 445 from 32 populations in IWP to analyze the genetic diversities and differentiations across sampling locations (**Table 1**) using the 18 SSRs in this study. To correlate the results with the geographic regions, we further categorized the populations into five geographic regions: the Philippine Archipelago (9 populations), Mainland Indo-China (5 populations in Vietnam and Thailand), the Malay Peninsula (the rest of the 10 populations in mainland Southeast Asia), Indonesia (1 population), and Sri Lanka (7 populations).

The percentage of polymorphic loci (%P, **Table 2**) ranges from 94% to 100% in the populations of Philippine Archipelago, from 89% to 100% in Mainland Indo-China and the Malay Peninsula, and 100% in Indonesia. Lower percentages of polymorphic loci were observed in some of the populations of Sri Lanka, 55% in three populations, while the rest of the populations in the region still show high %P (>80%). The overall percentage reaches ~92% across IWP. This confirms that the 18 SSRs are highly polymorphic and informative across the populations in this study.

**Table 2:** Genetic diversity indices of 32 nipa populations in the Indo-West Pacific using 18 SSR loci.

	Diversity indices^z^				
Population	Na	Ho	He	F	%P
**1. Philippine Archipelago (PH)**					
a. Mindanao South (PHMS)	4.667	0.259	0.616	0.583	100
b. Mindanao East (PHME)	5.111	0.252	0.670	0.649	100
c. Mindanao Northeast (PHMN1)	2.944	0.278	0.470	0.441	94.44
d. Mindanao North (PHMN2)	3.889	0.282	0.577	0.516	94.44
e. Central (PHCE)	5.278	0.285	0.655	0.577	100
f. Luzon East (PHLE)	4.722	0.307	0.635	0.548	100
g. Luzon West (PHLW)	4.000	0.265	0.534	0.521	100
h. Palawan West (PHPW)	3.556	0.306	0.600	0.556	100
i. Palawan East (PHPE)	4.500	0.370	0.626	0.421	94.44
**2. Mainland Indo-China (ML)**					
a. Vietnam 1 (MLVN1)	3.278	0.148	0.487	0.682	88.89
b. Vietnam 2 (MLVN2)	4.167	0.136	0.521	0.786	100
c. Thailand 1 (MLTH1)	3.222	0.056	0.455	0.837	94.44
d. Thailand 2 (MLTH2)	4.000	0.071	0.503	0.870	100
e. Thailand 3 (MLTH3)	3.667	0.057	0.491	0.846	100
**3. Malay Peninsula (MP)**					
a. Northern Coast 1 (MPNC1)	3.389	0.142	0.459	0.667	88.89
b. Northern Coast 2 (MPNC2)	3.556	0.101	0.521	0.823	94.44
c. Northern Coast 3 (MPNC3)	4.500	0.181	0.608	0.732	100
d. East Coast 1 (MPEC1)	4.611	0.217	0.543	0.616	100
e. East Coast 2 (MPEC2)	2.833	0.363	0.469	0.317	88.89
f. East Coast 3 (MPEC3)	3.667	0.167	0.537	0.737	94.44
g. East Coast 4 (MPEC4)	4.500	0.241	0.591	0.607	100
h. East Coast 5 (MPEC5)	3.389	0.144	0.512	0.711	94.44
i. Southern Coast (MPSC)	3.556	0.142	0.542	0.715	100
j. Southwest (MPSW)	4.222	0.178	0.587	0.692	100
**4. Indonesia-North Jakarta (INNJ)**	3.833	0.332	0.613	0.488	100
**5. Sri Lanka (SL)**					
a. Southwest 1 (SLSW1)	2.500	0.014	0.408	0.976	88.89
b. Southwest 2 (SLSW2)	2.167	0.005	0.372	0.989	83.33
c. Southwest 3 (SLSW3)	1.944	0.007	0.275	0.970	55.56
d. Southwest 4 (SLSW4)	2.444	0.021	0.403	0.956	83.33
e. West Coast 1 (SLWC1)	2.167	0.009	0.313	0.962	83.33
f. West Coast 2 (SLWC2)	1.611	0.000	0.195	1.000	55.56
g. West Coast 3 (SLWC3)	1.556	0.000	0.278	1.000	55.56
**Mean**	**3.545**	**0.167**	**0.502**	**0.697**	**91.67**
**SE**	**0.073**	**0.010**	**0.009**	**0.016**	**2.30**

z Na , number of different alleles; H**_o_**, observed heterozygosity; H**_e_**, expected heterozygosity; F, population’s fixation index; %P, percent polymorphic loci; SE, standard error. Mean: average of each parameter in all the populations. SE: Standard errors of the parameters in all populations. Boldface for the last two rows to distinguish the Mean and SE, respectively, from the raw data.

Genetic diversity of nipa populations in IWP was thus estimated by the following parameters using 18 SSRs: number of alleles (Na), observed (H_o_) and expected (H_e_) heterozygosity, and fixation index (F) (**Table 2**). We found that the populations in the Philippine Archipelago are the most genetically diverse with the highest Na particularly the central (5.278) and Mindanao East (5.111). Other populations with Na ≥ 4.500 can also be found in the Philippine Archipelago including Luzon East, Palawan East, and Mindanao South, and in the Malay Peninsula including Ko Yo, Mersing, and Terengganu North. Half of the populations in Sri Lanka had the lowest number of alleles between 1.556 and 2.500. In general, populations with a higher number of alleles show a higher percentage of polymorphic loci as well.

Lower observed heterozygosity than the expected heterozygosity was identified in all populations with mean H_o_ and H_e_ at 0.167 and 0.502, respectively (**Table 2**). This suggests that the populations are highly structured and differentiated across IWP and even within local populations. The mean fixation index (F) of all the local populations is 0.697, which indicates high differentiation of populations as well. Extremely high F indices were observed in the populations in Sri Lanka (0.97 to 1.0), possibly due to limited genetic connectivity with other geographic regions, which resulted in genetically isolated groups. Based on the above evidence, we may conclude that the populations in IWP are genetically diverged without frequent interbreeding between populations.

We further analyzed the genetic variations within and across populations by performing the analysis of molecular variance (AMOVA). The results showed that more than half (53.5%) of genetic variations were contributed by the variations between individuals within populations, while 22.1% and 24.4% of variations are among populations and within individuals, respectively (**Table 3**).

**Table 3:** Analysis of molecular variance (AMOVA) of nipa populations across the Indo-West Pacific using 18 SSR loci.

Source of variation	Sum of Squares	Variance components	Percentage variation	P-value
				
Among populations	1472.411	1.42028	22.13118	0.00000*
Among individuals within populations	3459.124	3.43047	53.45440	0.00000*
Within individuals	693.500	1.56681	24.41442	0.00000*
Total	5625.035	6.41757		

*Significant at P < 0.05.

This genetic pattern was also reflected by F-statistics across all loci, which are the indices of population differentiation. The fixation indices serve as an expansion to describe population structure at different levels: inbreeding coefficient of individuals within subpopulation (F_IS_), inbreeding among subpopulation within the total population (F_ST_), and overall inbreeding coefficient (F_IT_). The results show very high overall inbreeding coefficient with that of within population (F_IT_ = 0.7559 and F_IS_ = 0.6865), while lower inbreeding coefficient was detected between populations (F_ST_ = 0.2213) relative to other indices. This result confirmed much lower observed heterozygosity in all the populations mentioned above. Taken together, the results of AMOVA and F indices indicate that the excess of homozygosity shapes the populations in IWP.

The BayeScan results detected balancing selection signatures for most of these SSR loci as determined by a negative alpha value and a Q-value greater than 0.05. Eleven out of 18 were balancing loci, and 7 were considered neutral (**Table S2**). If critical level was set for Q-value = 0.05, then Nfr58, Nfr60, and Nfr69 had significantly strong balancing selection for local adaptation. Generally, population differentiation under balancing selection will be weaker than neutral loci as observed in self-incompatible plants (Fijarczyk and Babik, 2015). These loci obtained the lowest F_ST_ values (0.23093, 0.20837, and 0.18678, respectively) compared to neutral loci (0.335–0.362) (**Table S2**), which supported the balancing signal. Neutral loci include Nfr14, Nfr17, Nfr22, Nfr27, Nfr30, Nfr40, and Nfr56. There were no outliers for diversifying loci as an indication of positive selection. Among the signatures of balancing selection enumerated by Fijarczyk and Babik (2015) as temporal and may have significance for the sets of loci in this study include increased LD and differentiation among populations. We cannot rule out the so-called selective sweep as responsible for recent increases in homozygosity detected due to balancing selection signature.

The analysis of MLG showed that populations in Southeast Asia (ML and MP) were more genotypically diverse than those of Sri Lanka (**Table S3**). We observed higher MLG than expected (eMLG) in most of the populations in the Philippine Archipelago, Mainland Indo-China, and the Malay Peninsula. Almost equal number of MLG and eMLG were found in Indonesia and Sri Lanka. The result suggests a lower genetic diversity in the populations of Sri Lanka, which might be caused by several factors.

We further tested whether the populations were under LD [estimated by the values of rbarD (r̄_d_) in **Table S3**], which likely resulted from nonrandom mating (clonal reproduction) or genetic drift. Together with the index of association (I_A_), we can tell whether the clonal reproduction is the possible factor causing populations away from Hardy–Weinberg equilibrium. Among all the populations, only three of those showed evidence of Hardy–Weinberg equilibrium for the loci tested (*p*.rD > 0.05, **Table S3**). They are Mindanao-Northeast (PHMN1), Palawan East (PHPE), and Negombo (SLWC1) populations, two in the Philippine Archipelago and one in Sri Lanka. In addition, the three populations showed equal number of MLG with eMLG, indicating that they might be panmictic populations and putative centers of origin (Grünwald et al., 2017). Furthermore, nipa has clearly sexual populations since observed MLG counts are mostly equal to respective sample sizes (N) (**Table S3**). Clonal populations have greater N than MLGs because of duplicates (Kamvar et al., 2014).

### Significant genetic divergence detected between individuals when they are more than 1,000 km apart

**Figure 1:**
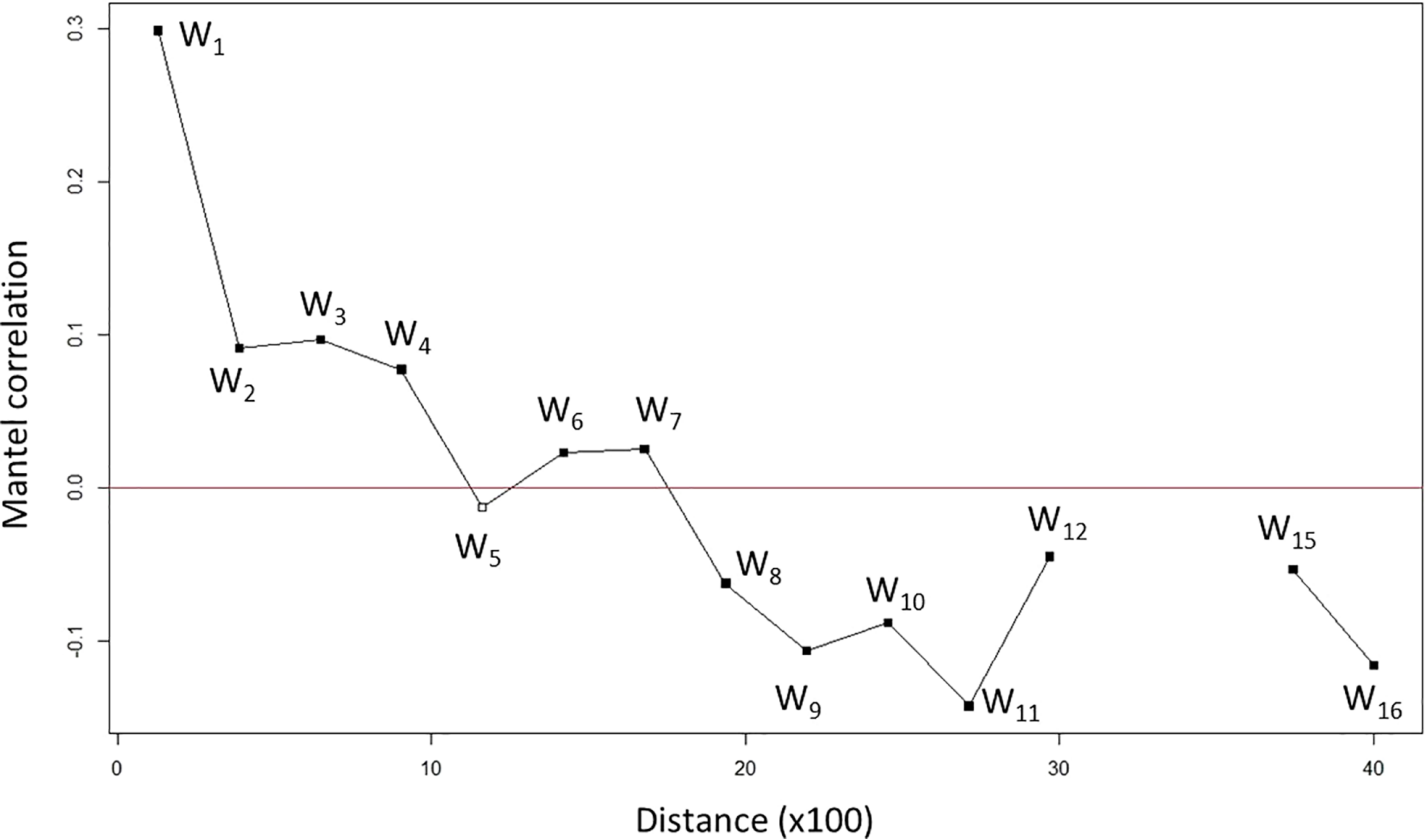
Genetic divergence increased (correlation decreased) along with the geographic distances of nipa populations across IWP. The plot is generated by a Mantel correlogram indicating distance (×100) in kilometers. For example, 10 means 1,000 km apart. Black solid squares show significant correlation while a white empty square shows no significance in correlation value. Each distance class is labeled as W_k_ (*k* = 1 to 16 with 13–14 no correlation).

The effect of isolation-by-distance (IBD, Mantel test) was statistically significantly detected (*p* < 0.001 in *n* = 1,000) with a positive correlation (*r* = 0.4199), which indicates a higher genetic divergence between two individuals when they are farther apart. A Mantel correlogram was generated to further analyze the relationship between genetic divergence and geographic distance (**Figure 1** and **Table S4**). It shows the relationship between genetic divergence (represented by correlation, *r*) and geographic distances across geographic regions. We conducted Mantel correlogram tests. As an extension of the Mantel test, a correlogram can show the relationship between G and D matrices across geographic regions (Diniz-Filho et al., 2013).

We observed a decreasing correlation from the first to the fifth class, W_1_–W_5_ (about 1,000 km distance), where the correlation of W_5_ turned negative (**Figure 1**). This implies that a pair of samples has no genetic correlation when they are about 1,000 km apart. At W_8_ (about 2,000 km apart), a pair of samples was differentiated with significant negative correlation. Although an increase in correlation was observed in W_12_ (about 3,000 km apart), it is still negative. In fact, it was abruptly terminated beyond that point. There were no observations between W_13_ and W_14_ (within a 1,000-km range), indicating no spatial correlation. Taken together, the Mantel correlogram spatial analysis suggests a significant genetic divergence of individuals when they are more than 1,000 km apart geographically. This supports our previous observations, i.e., IBD and structured populations with geographical patches of nipa in IWP.

### Distinct genetic clusters between Southeast Asian and Sri Lankan populations with the Malay Peninsula as a possible genetic barrier

**Figure 2:**
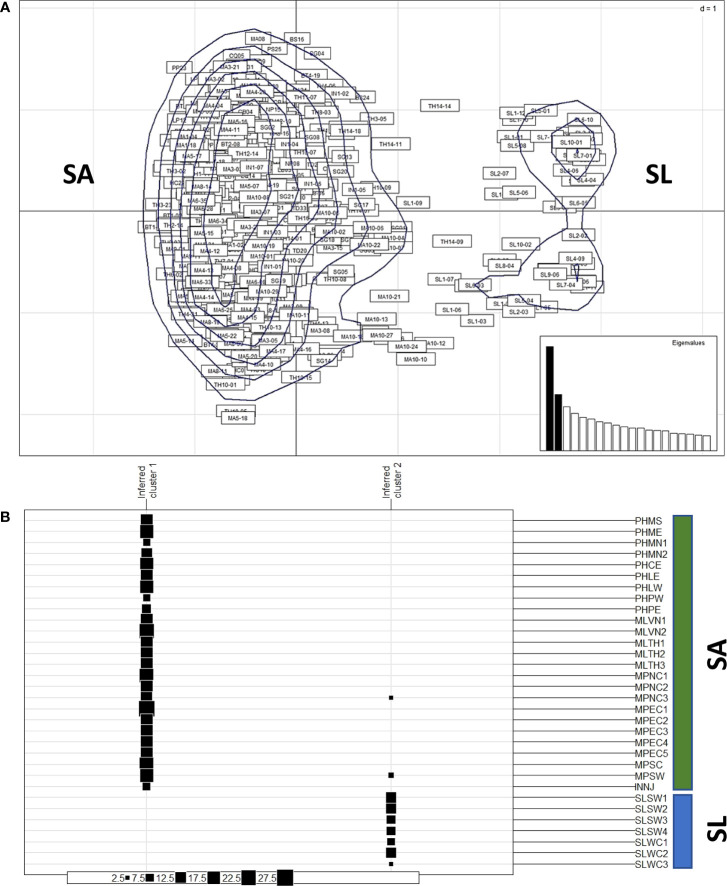
Clustering of populations from Southeast Asia (SA) and from Sri Lanka (SL). **(A)** The principal coordinates analysis (PCoA) of nipa samples from 32 populations in IWP showing the kernel density estimate of individuals, which can be clustered into two major groups: one larger group includes samples from SA and one smaller cluster includes samples from SL. **(B)** Discriminant analysis of principal components (DAPC) suggests two clusters (K = 2) of nipa populations in IWP according to inferred clusters. The group size of each location is indicated by solid boxes (from 2.5 to 27.5). The symbols of locations are according to **Table 1**.

The distribution of the 445 nipa samples across 32 locations in IWP appeared to group into two clusters based on PCoA (**Figure 2A**). The major group includes the populations of mainland Southeast Asia and Indonesia, while the minor group includes populations of Sri Lanka. The multivariate DAPC, an alternative method to analyze genetic pattern, also showed similar clustering of samples (**Figure 2B**). Two major clusters from Southeast Asia were grouped in one cluster (cluster 1), while those from Sri Lanka were in a distinct separate cluster (cluster 2). Interestingly, we identified two individuals grouped with Sri Lanka from the Malay Peninsula (MPNC3 and MPSW), which were likely effective migrants or hybrids between the two regions.

Although principal component analysis (PCA) has been used as a common multivariate approach in population genetics, it does not explicitly account for spatial genetic patterns (Montano and Jombart, 2017). A novel approach, sPCA, determining the spatial structure as measured by Moran’s index and eigenvalue, was conducted to further examine the genetic patterns of nipa populations. The positive values of Moran’s index indicate the high similarities of allele frequencies in the neighboring regions and may indicate globally structured populations in distant regions. In contrast, the negative values indicate genetic divergence and locally structured populations.

**Figure 3:**
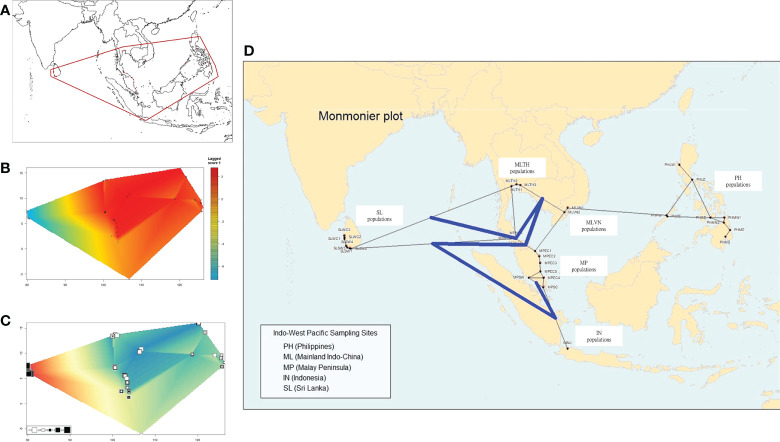
Spatial correlations and genetic differentiation of nipa populations based on the sPCA method. **(A)** Distribution of sampling locations, which serves as the range used in the following spatial analysis in **B–D**. **(B)** Overall genetic divergence and spatial connectivity of populations. The color bars on the right indicate the degree of population structure: the dark red means the populations are globally structured while the blue indicates no similarity between two individuals (extremely locally structured). **(C)** The first axis (PC) of the sPCA plot. **(D)** The Monmonier plot of genetic boundaries (solid blue line) shows that the Malay Peninsula is a genetic barrier among the connection network of nipa populations in IWP.

Overall, a strong spatial connectivity was detected among populations in mainland Southeast Asia (**Figure 3A**). This connectivity is represented by a colored background based on the lagged scores of populations across landscape (**Figure 3B**). A strong connectivity network was observed around the South China Sea (SCS) based on a dark red background. Falling outside the red edges on yellow orange and orange background were the populations from the Southern Coast of the Malay Peninsula (MPSC) and the Southwest Coast of the Malay Peninsula (MPSW), along the Malacca Strait, and further south, Indonesia. This indicates that they were less associated with the rest of the populations around SCS with some degree of genetic divergence. The populations of Sri Lanka were distinctly separated from mainland Southeast Asia, plotted along the leftmost edge of the polygon, and are in shades of blue (**Figure 3B**).

The first PC clearly shows the distinctions between the west (Sri Lanka) and the rest of the other populations (Southeast Asia group) on shades of green background (**Figure 3C**). The values, as represented in squares, indicate that the populations in Southeast Asia had subtle genetic divergence and likely a clue of a possible population differentiation. Moreover, Monmonier’s maximum difference algorithm was implemented to evaluate a genetic barrier (**Figure 3D**). It identifies boundaries in the areas where differences between a pair of populations are the greatest (Manni et al., 2004). The result showed that the Malay Peninsula is a genetic barrier as indicated by a solid blue line. It is worth mentioning that there were some individuals from the Malay Peninsula (MPNC3 and MPSW) grouped with the individuals from Sri Lanka based on DAPC (**Figure 2B**). These individuals might likely be effective migrants or hybrids between the two clusters. However, the hybridization between the two clusters was not detected further as the potential genetic barrier of the Malay Peninsula.

### Subpopulation structures in SA and SL

**Figure 4:**
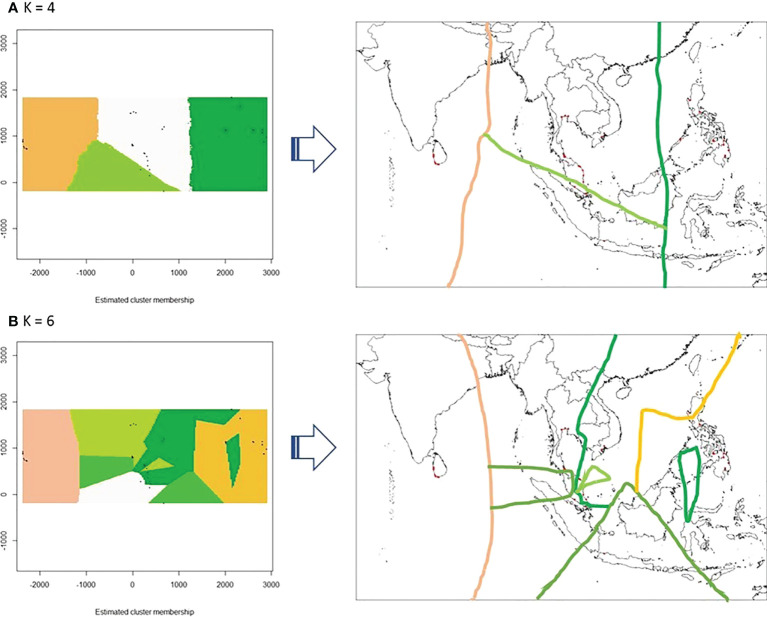
Population differentiations of nipa analyzed using the *Geneland* package **(A)** The spatial model (K = 4) and **(B)** nonspatial model (K = 6) for nipa populations in the Indo-West Pacific showing genetic discontinuities as interpreted geographically (arrow).

A more detailed genetic divergence can be analyzed using the package *Geneland* under the spatial model. The results show that the best clustering (K) was obtained in four genetic clusters (**Figure 4A**). The populations of the Philippine Archipelago belong to one group (dark green), while those in mainland Southeast Asia and part of the Malay Peninsula were grouped together (white). The populations from Negeri Sembilan (MPSW), the only west coast population in the Malay Peninsula, Singapore (MPSC), and Indonesia (INNJ), belonged to the same group (light green). Lastly, Sri Lankan populations were distinctly separated from the other genetic clusters (orange).

Under the nonspatial model, the best K was outright at 6 without the necessary convergence of MCMC iterations (**Figure 4B**). Some populations such as Luzon West (PHLW) and Palawan East (PHPE) in the Philippine Archipelago clustered with mainland Southeast Asia. Although the populations in Southeast Asia are more structured under this model, the populations in Sri Lanka remained genetically distinct.

**Figure 5:**
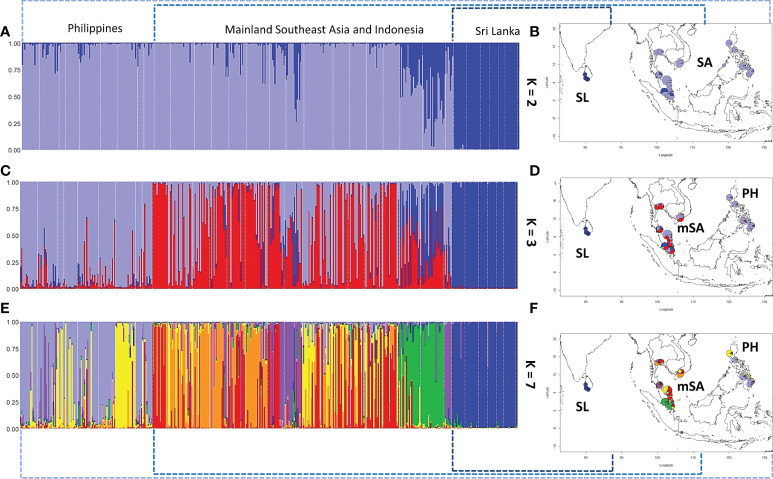
The population differentiation and genetic clustering using the Evanno method in STRUCTURE. Samples from different populations can be clustered into two, three, or seven groups. **(A, B)** Samples are clustered into two groups: SL represents Sri Lanka and SA represents Southeast Asia. **(C, D)** Samples are clustered into three groups. Under this condition, populations in SA show differentiation between mainland SA (mSA) and the Philippines (PH). **(E, F)** When seven groups were set, more detailed differentiations can be identified within mSA and PH.

Using the Evanno method in STRUCTURE, the best genetic clustering of all populations is 2 (K = 2): populations in Sri Lanka were distinctly separated from Southeast Asia (**Figures 5A, B**). When three clustering groups were set (K = 3), differentiations between populations in the Philippine Archipelago and mainland Southeast Asia were observed (red color in **Figures 5C, D**). We found that the populations in mainland Southeast Asia and the Philippine Archipelago can both further be grouped into several subclusters: the Philippine Archipelago, Mainland Indo-China (Vietnam and Thailand), the southern Malay Peninsula, and Sri Lanka (**Figures 5E, F**). In particular, the populations in mainland Southeast Asia showed differentiations to a certain degree under this clustering condition.

Taken together, we conclude the two tiers of genetic differentiations: (1) all the analyses show the solid evidence of isolated and differentiated populations in Sri Lanka compared to other populations in Southeast Asia; (2) populations in the Philippine Archipelago are differentiated from those in mainland Southeast Asia.

### Migration may have shaped the phylogeography of nipa populations in IWP

**Figure 6:**
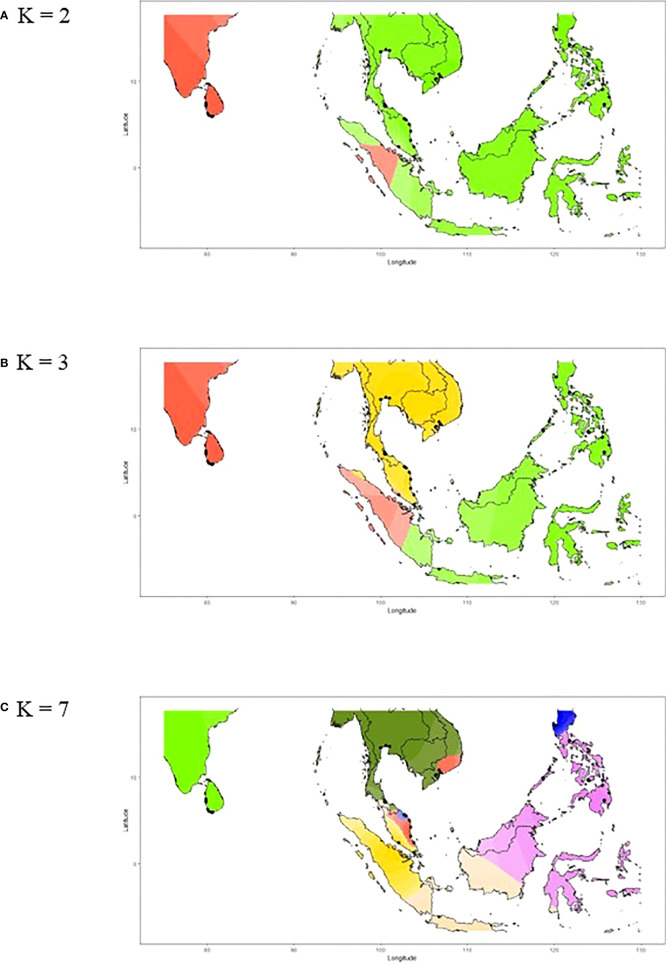
The ancestry coefficients of nipa individuals across the Indo-West Pacific. **(A)** K = 2, **(B)** K = 3, and **(C)** K = 7 based on the individual Q-matrix of STRUCTURE as interpolated by R using *tess3r*.

We further investigated whether the connectivity of the populations between Sri Lanka and Southeast Asia exists despite the fact that they are geographically isolated without active breeding. The ancestry coefficient analysis showed that the genealogical lineages of nipa populations can be categorized into two clusters, i.e., Sri Lanka and Southeast Asia for K = 2 (**Figure 6A**). Populations in Southeast Asia can be further subdivided into mainland Southeast Asia and the Philippine Archipelago where Indonesia (INNJ) shared ancestry when K = 3. The island of Sumatra was projected to share similar ancestry with Sri Lanka (light orange in **Figure 6B**). Take one step further to set K = 7, we observed that the genetic divergence of populations from Luzon West (PHLW, blue in **Figure 6C**) and Vietnam 1 (MLVN1, orange in **Figure 6C**) shared putative ancestry with the populations in the southeastern coast of the Malay Peninsula (**Figure 6C**). Taken together, we proposed that migrations have happened in the following two trends: (1) between populations of Sri Lanka and Southeast Asia, particularly the island of Sumatra, and (2) between populations of the Malay Peninsula, mainland Southeast Asia, and the Philippine Archipelago. This also in line with evidence that the Malay Peninsula is the genetic barrier of nipa populations in IWP as mentioned above.

The founder effect is expected if the individuals migrated from mother populations, such as Southeast Asia, to a new niche further away. Therefore, the reduction of genetic diversity of new founders compared to the mother population due to genetic drift is expected. Based on the graphical descriptor, Sri Lankan populations were all “mode-shifted”, meaning these stands experienced bottleneck recently or sometime in the past (**Table 5**). Except for Balapitiya (SLSW1), all southern populations were detected significant for the one-tailed Wilcoxon’s test for heterozygosity excess indicating recent bottlenecks. For the west coast populations, only Puttalam (SLWC3), being depauperate, was tested to experience it recently. The so-called mode-shifted populations not significant to all Wilcoxon’s tests might have been bottlenecked in the distant past, likely a consequence of the founder effect.

**Figure 7:**
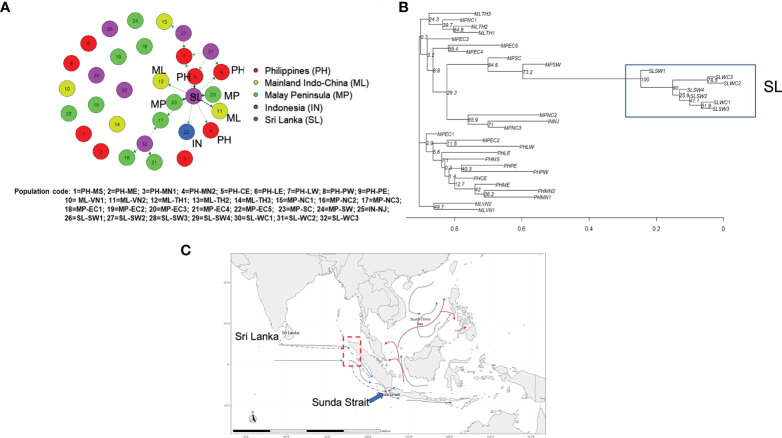
Migration and phylogeny of nipa populations in IWP. **(A)** The contemporary migration patterns of nipa populations in the Indo-West Pacific using the *divMigrate* function of diveRsity, an R statistical package, for the significant relative migration based on *Nm* using filter threshold at 0.12 for asymmetric values (bold and dark arrows have higher migration values than light colored arrows) (bootstrap = 1000). **(B)** Phylogenetic tree constructed with 18 microsatellite loci using the neighbor-joining (NJ) method (bootstrap = 1000). **(C)** The hypothesized migration pattern from Sri Lanka (dashed red arrow) *via* the hypothetical route to northwestern Sumatra nipa populations (red dashed line and dash-lined box) by long-distance dispersal to Southeast Asia through Sunda Strait as labeled accordingly. The blue arrow lines indicate the predominant surface current during the Southwest Monsoon (Schott and McCreary, 2001; Han et al., 2009; Wang and Li, 2009; Susanto et al., 2016) and the red arrows indicate the significantly strong patterns in migration network results.

To graphically evaluate the genetic clusters of nipa populations in the region, a phylogenetic tree was reconstructed by the neighbor-joining (NJ) method. The result again indicated the genetic distinctiveness of populations from Sri Lanka, which was further clustered with a population from the Malay Peninsula, MPSW. It is in line with the finding from DAPC (**Figure 2B**) where some individuals from MPNC3 and MPSW in the Malay Peninsula were clustered with Sri Lankan populations. This further confirms the genetic connectivity between Southeast Asia and Sri Lanka in certain populations. The tree is represented by the unrooted phylogram as shown in **Figure 7B**. Notably, the order of the clustering was uncertain because of extremely low bootstrap support at the deeper nodes.

Notwithstanding the lines of evidence of the founder events in Sri Lanka by oceanic circulations, there are indications of stronger genetic link eastward than westward, i.e., Matara (SLSW3) to the Southeast Asian populations based on the contemporary genetic diversity (**Figure 7A** and **Table S5**). We consider this pattern as recent migrations where interbreeding with local populations in Southeast Asia diminished their ancestral genetic lineage that resulted in low bootstrap support (**Figure 7B**). We, therefore, proposed the recent migration routes of nipa and hypothesized that their potential entry point from Sri Lanka to SCS might be the Sunda Strait (**Figure 7C**).

## Discussion

Nipa remains an understudied mangrove palm in spite of known economic and ecological benefits. It was considered as a potential bioenergy crop, but has no breeding program and agronomic practices in place. To fill in the gap of limited information on its population genetics, the evaluation of genetic diversity and structure was conducted in the IWP Region.

All populations in the region reflected a genetic pattern, suggesting some factors or biological processes acting on them. High discrepancies between observed heterozygosity (H_o_) and expected heterozygosity (H_e_), as supported by relatively high F_ST_ along with balancing selection signature, indicate an excess of homozygosity due to admixture. Test for linkage equilibrium using the standardized index of association (r̄_d_) was significant, indicating mostly non-panmictic populations resulting in LD. Also, lower expected multilocus genotype (eMLG) values compared to observed MLG were posited to be enforced by admixture. The mating pattern of nipa as outcrossing possibly resulted in this genetic pattern in the region from LD by migration. As a consequence, homozygosity was higher than expected with random mating. This is called the Wahlund’s principle (Hartl, 2020). This genetic pattern was observed across the local populations based on heterozygosity values, H_o_ and H_e_, and F.

Genetic assignment of first-generation (F_0_) immigrants (**Table S5**) detected over half of them in mainland Southeast Asia, enriching these populations aside from genetic exchanges from nearby populations. These biological processes shaped the local populations to deviate from Hardy–Weinberg equilibrium that results in the population structure in the region. In addition, the Mantel test showed significance for IBD. The kernel density estimate indicates patches instead of an intact cloud, suggesting genetic discontinuity (figure not shown). A systematic change in allele frequency across a geographical gradient is called a cline (Hartl, 2020). These genetic differences are not merely affected by geographic cline, but by isolation. Apparently, there was a clear genetic differentiation between the west populations, i.e., Sri Lanka and Southeast Asia, due to limited genetic exchange, which unanimously attributed this population stratification in the region based on several tests.

Although genetic clines were also detected among the populations in Southeast Asia, the SCS remains a hotbed of genetic exchange among nipa populations. Those stands found along its coasts had stronger genetic exchanges where the southeast Malay Peninsula was thought to be a contact zone for admixture. As a melting pot, immigrants were shown to have their genetic ancestries coming from far-flung populations such as Sri Lanka, Indonesia, and the Philippines. Moreover, the spatial genetic pattern suggests that populations closer to each other consisted of individuals that are closely related too. Moreover, Singapore and Negeri Sembilan along the Malacca Strait showed signs of genetic differentiation as well as that of Indonesia at the coast of the Java Sea. The population stratification supports the biogeographic subregions of Wallace, which he proposed in 1876 (Woodruff, 2010) except that the boundary in the Malay Peninsula was pushed further south as likely shaped by contemporary physical processes. This was highly attributed to the predominant seasonal monsoon reversal along with the oceanic circulations as a means of seed dispersal.

In addition, evaluating genetic relationships among populations of species in its distribution range can be best understood by phylogeography. It has been a fast, emerging field involving the analyses of geographic distributions of genealogical lineages, especially those within species level. The statistical and mathematical aspects of phylogeography were a recent innovation of what is now called coalescent theory where genealogies are addressed through space and time (Avise, 1998). While tests are often conducted in tandem with matrilinear chloroplast (cp) DNA among plants, microsatellites can nevertheless serve as stand-alone markers. For instance, using microsatellites alone, Charrier et al. (2014) detected two lineages of the subalpine shrub, *Rhododendron ferrugineum*, that colonized Pyrenees from the Alps. This species survived in different refugia during glaciation events, resulting in a clustered genetic pattern. The Pyrenees, a mountain range between France and Spain, acted as a biogeographical barrier during the postglacial expansion. Among mangrove species, Wee et al. (2014) employed 10 microsatellite loci to assess the phylogeography of *Rhizophora mucronata* in Southeast Asia.

The so-called North Equatorial Current that dominates the Central Pacific has, first and foremost, an impact in the Philippines. It is brought about by trade winds that produce a primary current pushing the water from east to west. It partly flows down south to the Celebes Sea, but mostly flows north called the Kuroshio. Moreover, the flow of water around Southeast Asia and as far as Northern Australia is influenced largely by the monsoon wind. Around November to April, the cool continental air called the Northeast Monsoon flows to a relatively warmer Indian Ocean (IO). The flow in the SCS is predominantly southwest, then the direction reverses around May to October. The cool ocean air called the Southwest Monsoon flows mostly over the SCS in a northeast direction (Carpenter and Niem, 1998).

The surface currents in the IO follow the patterns of other oceans, but during Northern Hemisphere (boreal) summer, the westward North Equatorial Current is replaced and the weaker Equatorial Countercurrent merges with the Southwest Monsoon Current flowing eastward. It curves in a clockwise direction near Sumatra that flows westward, and circulates clockwise north of the IO. The Somali Current and Equatorial Jet influence northward and eastward surface circulations, respectively, during the seasonal monsoon changes (Bowditch, 2002). These monsoon reversals appeared to have influenced the migration patterns in Southeast Asia that allowed these genetic relationships between the local populations in spite of their distances.

The rates of gene flow can be estimated using Slatkin’s private allele method using the number of migrants (*Nm*) as the effective number of migrants per generation. It was assumed that the private alleles unique to a population are likely to attain high frequency only when *Nm* is low (Ellsworth and Hoelzer, 2006). In short, the presence of private allele in one population is indicative of no migration (Sundqvist et al., 2016). *Nm* was estimated from the frequencies of private alleles with correction for differences of samples sizes of populations. By the simplest migration island model, values of *Nm* greater than 1 indicate the homogenizing influence of gene flow, while a value as high as 6 can permit substantial divergence under the model IBD based on simulation studies (Ellsworth and Hoelzer, 2006). The overall migration rate of nipa populations in the region was considered marginal, *Nm* = 1.09272 (**Table 4**). For other mangroves, the overall *Nm* of *Bruguiera gymnorrhiza* and *Kandelia obovata* in Okinawa, Japan, were estimated to be 1.826 and 4.681, and the *p* (l) or mean frequency of private allele was 0.049 and 0.029, respectively (Basyuni et al., 2018).

**Table 4:** Number of migrants (*Nm*) using private alleles.

Sample size	*Nm*
Mean *N* = 10	1.51121
Mean *N* = 25	0.751965
Mean *N* = 50	0.524906
Corrected value for sample size	1.09272

Mean sample size: 13.8299.

Mean frequency of private alleles, p (1): 0.0914766.

Also, F_ST_ is an indirect measure of migration rate with the following expression: F_ST_ = 1/(1 + 4*Nm*). *Nm* is inversely related to F_ST_, such that when *Nm* is large, F_ST_ is small and *vice versa*. However, Whitlock and McCauley (1999) dismissed this mathematical equation as F_ST_ can rarely estimate *Nm* accurately. Nevertheless, Yamamichi and Innan (2012) argued that when assumptions of island model, like neutrality, are violated, *Nm* is most sensitive to recent migration, while F_ST_ is most sensitive to migration that occurred over a relatively longer time as it involves common alleles. With highly variable markers like microsatellites, it is apparent that there was population subdivision at the level of F_ST_ = 0.22131 (**Table 5**) in conjunction with migration rather than population divergence. This must be validated further as to how dispersal affects genetic differentiation among subpopulations in the region.

**Table 5:** All the p-value of Wilcoxon’s tests for bottleneck of nipa populations in the Indo-West Pacific across 18 microsatellite loci^z^.

Population	Wilcoxon 1-tailed TPM	Wilcoxon 2-tailed TPM	Wilcoxon 1-tailed SMM	Wilcoxon 2-tailed SMM	Mode-shift
PHMS	0.5507	0.9323	0.7101	0.6095	Shifted
PHME	0.1519	0.3038	0.2613	0.5226	Normal
PHMN1	0.6090	0.8176	0.6090	0.8176	Shifted
PHMN2	0.1123	0.2247	0.1421	0.2842	Normal
PHCE	0.3353	0.6705	0.5339	0.9661	Normal
PHLE	0.1419	0.2837	0.2613	0.5226	Normal
PHLW	0.1733	0.3466	0.3047	0.6095	Shifted
PHPW	**0.0152**	**0.0304**	**0.0192**	**0.0385**	Shifted
PHPE	0.1764	0.3529	0.2153	0.4307	Shifted
MLVN1	0.0795	0.1591	0.1742	0.3484	Normal
MLVN2	0.6491	0.7337	0.7789	0.4683	Normal
MLTH1	0.4450	0.8900	0.5732	0.8900	Normal
MLTH2	0.9962	**0.0090**	0.9986	**0.0034**	Normal
MLTH3	0.9667	0.0737	0.9896	**0.0237**	Normal
MPNC1	0.4699	0.9399	0.5699	0.8999	Normal
MPNC2	**0.0116**	**0.0232**	0.0544	0.1089	Normal
MPNC3	**0.0407**	0.0814	0.1144	0.2288	Normal
MPEC1	0.9093	0.1964	0.9551	0.0987	Normal
MPEC2	**0.0003**	**0.0005**	**0.0004**	**0.0008**	Shifted
MPEC3	**0.0075**	**0.0150**	**0.0284**	0.0569	Normal
MPEC4	0.8267	0.3692	0.8769	0.2645	Normal
MPEC5	**0.0253**	0.0505	0.0601	0.1202	Normal
MPSC	0.0837	0.1674	0.1323	0.2645	Shifted
MPSW	0.0770	0.1540	0.1323	0.2645	Normal
INNJ	**0.0152**	**0.0304**	**0.0407**	0.0814	Shifted
SLSW1	0.0964	0.1928	0.1261	0.2522	Shifted
SLSW2	**0.0051**	**0.0103**	**0.0051**	**0.0103**	Shifted
SLSW3	**0.0420**	0.0840	**0.0420**	0.0840	Shifted
SLSW4	**0.0206**	**0.0413**	**0.0277**	0.0554	Shifted
SLWC1	0.8853	0.2524	0.8853	0.2524	Shifted
SLWC2	0.2461	0.4922	0.2158	0.4316	Shifted
SLWC3	**0.0005**	**0.0010**	**0.0005**	**0.0010**	Shifted

z Highlighted rows and in boldface indicate significance (p < 0.05) in tests either 1-tailed (for heterozygosity excess) or 2-tailed (for heterozygosity excess or deficit) under two-phase mutation (TPM) or stepwise mutation model (SMM). Mode-shift is a graphical descriptor of the allele frequency distribution which distinguishes bottlenecked over stable populations.

The strong genetic connectivity from west to east does not necessarily indicate that Southeast Asia is not a corridor for migration. In fact, this dispersal to Sri Lanka was detected (**Figure 4**). It was likely a weak signal indicating non-significance in the network statistically. Bowditch (2002) pointed out that the eastern boundary of the IO was known to have limited interaction with the PO. By and large, this is acted upon by the SCS as an extension of the PO (Qiu et al., 2015). In fact, the Monmonier plot showed that the Malacca Strait is a genetic barrier (**Figure 3D**). Being a narrow sea passage between southwest of the Malay Peninsula and Sumatra, it limits, if not restricts, a southern migration route from the SCS to the IO. This confirms various reports that the Malacca Strait was not only a genetic but also a geographic barrier, among mangrove species (Mantiquilla et al., 2021). For nipa, this remains to be understood as the study had few sampling sites along the Malacca Strait, and particularly none in Sumatra, i.e., the island across the west coast of the peninsula. In this case, gene flow could also possibly occur somewhere else.

The SCS and the PO strongly influenced the water circulations within the Philippine Archipelago. The westward-flowing water current from western PO reverses direction with season. Interestingly, the Bohol Sea current reverses eastward during the Southwest Monsoon (Han et al., 2009). Those effective migrants may have taken along the routes of western inflows across the SCS, then to central Philippines by the direction reversal of the Bohol Sea. Other Sri Lankan populations that were detected to have a migration pattern throughout Southeast Asia, albeit moderately, include Dondra (SLSW2), Nainamadama (SLWC2), and Puttalam (SLWC3).

While directional gene flow showed that Sri Lanka as a source was more common in the migration network, it is instinctive to assume that many transoceanic dispersal events to this island might have occurred, but nevertheless unsuccessful. Voyages westward appeared to have propagules selected against time. In fact, recent data of the surface current of the Northeast Monsoon was observed to be always weaker than that of the Southwest Monsoon because of the weaker associated winds based on 2010–2012 data. The transport rate was measured in Sverdrup (Sv), a unit of flow equivalent to 1 × 10^6^ m^3^ s^−1^, as follows: Northeast Monsoon Current obtained a mean of 9.6 Sv with a range of 8.27–10.69 Sv compared to Southwest Monsoon Current estimated at 11.5 Sv (range: 10.25–12.78 Sv) (de Vos et al., 2014). Consequently, the time is ticking for these immigrants being washed ashore and settled in favorable habitats affecting their chances of survival. If so, then the test for bottleneck may provide some clues on the fate of these dispersals in the past.

Based on the constructed phylogenetic tree, all populations in the deeper nodes had extremely low bootstrap support. The first group, the largest and covered the widest geographically, consisted of Thailand and most of Malay Peninsula populations. Among the populations, Bang Riang (MPNC2) and Ko Yo (MPNC3) shared the same branch with Indonesia (INNJ), which was monophyletic with southern populations Singapore (MPSC) and Negeri Sembilan (MPSW). In particular, the sister branch of MPSW that bears the longest branch was the clade of Sri Lankan populations (**Figure 7B**). The length of this branch suggests a considerable time that elapsed prior to the emergence of this dichotomy. Nevertheless, the bootstrap support for the ancestral population of Sri Lanka clade was robust at 100%. At a bootstrap of 1,000, a score of 90% indicates pretty high confidence on the reliability of the clade (Hall, 2008). Indeed, this clade with Balapitiya (SLSW1) at the basal node was fairly recent relative to other populations in the region. This supports the tests for bottleneck that a founder event involving SLSW1 may have occurred sometime in the distant past. Another founder event might have occurred involving Nainamadama (SLWC2) or Puttalam (SLWC3) very recently as indicated by a node in the clade. The rest of the other nodes were seen as a range expansion at the distribution edge. Because of the complex oceanic circulation in the IO, the paucity of success among dispersal events likely caused genetic drift in these marginal populations.

The general circulations of the eastern boundary of the IO involve the northbound and southbound surface water masses that flow along the Andaman Sea in both monsoons. Particularly during the Northeast Monsoon, the southbound surface current flows down to Sumatra and then to the IO. At this time, Rizal et al. (2012) also observed that the presence of an anticlockwise gyre, and a northbound current during the Southwest Monsoon, consistently blocked the outflow of the Malacca Strait. Wee et al. (2014) attributed to these surface currents the genetic patterns of *R. mucronata* populations in Southeast Asia using 10 microsatellite loci. The north Sumatra populations, although closer to the populations along the Malacca Strait, were genetically clustered with the Myanmar population at the relatively distant northwest coast of the Malay Peninsula. Moreover, the ocean simulations of these findings also found out that the Malacca Strait was indeed a geographical barrier, confirming what was reported elsewhere.

Nipa populations in Sumatra, Indonesia, are unquestionably important, which may shed light on the link of the gene flow in east–west subdivision in the IWP. Inventory of the sampling sites on the post-tsunami (2004) impact on mangrove populations showed that nipa remained standing by sheer numbers both in the north (Banda Aceh) and along the west coast (Aceh Barat and Nias Barat) of Sumatra (Onrizal and Mansor, 2016).

All nipa populations in the Philippine Archipelago were paraphyletic to Terengganu Central (MPEC2) and polyphyletic to the more ancestral Terengganu North (MPEC1) population (**Figure 7B**). Again, bootstrap support for these populations was generally low, but their affinity provides a clue for dispersal. The ocean circulation serves as a corridor for emigrants from Luzon East to Terengganu by the intrusion of Kuroshio to the SCS. However, Sundqvist et al. (2016) reasoned that historical demography may influence the directional migration such that it may enhance or, in this case, diminish signs of recent migration. Consequently, the genetic footprints of these ancestral populations may have also been obscured by the admixture of recent genetic ancestry resulting in poor bootstrap support in the phylogram. This requires an alternative higher-resolution marker to elucidate the answer.

Given this information, Southeast Asia remains the center of diversity of nipa as this subregion is predominantly influenced by oceanic circulations in many directions. These physical processes continuously provide immigrants that enrich the local populations. The populations in Sri Lanka, on the other hand, were considered as recent expansion at the margin of the distribution range, estimated between 250 ka and 150 ka. Rapid succession of this expansion was observed during the LGM, and was consistent with the bottleneck signatures. Characterized as having low genetic variation with plausible genetic drift in the past, these peripheral populations will likely be pushed to extinction if not given proper attention.

## Data availability statement

The original contributions presented in the study are included in the article/**Supplementary Material**. Further inquiries can be directed to the corresponding author.

## Author contributions

Conceived and designed the review: JM and Y-CC. Field sampling: JM, M-SS, H-YL, KS, SS, WL, Y-LC, and Y-CC. Writing and figures: JM, M-SS, H-CS, and Y-CC. Final proofreading and editing: JM, M-SS, and Y-CC. All authors contributed to the article and approved the submitted version.

## Funding

This paper was supported by funding from the Ministry of Science and Technology, Taiwan (MOST 108-2621-B-110-003-MY3, MOST 109-2313-B-110-005, and MOST 111-2621-B-110-001) to Y-CC and by partial financing (the Higher Education Sprout Project) of NSYSU.

## Conflict of interest

The authors declare that the research was conducted in the absence of any commercial or financial relationships that could be construed as a potential conflict of interest.

## Publisher’s note

All claims expressed in this article are solely those of the authors and do not necessarily represent those of their affiliated organizations, or those of the publisher, the editors and the reviewers. Any product that may be evaluated in this article, or claim that may be made by its manufacturer, is not guaranteed or endorsed by the publisher.

## Abbreviations

SCS, South China Sea; PO, Pacific Ocean; IO, Indian Ocean.
