# Supplementary material for: Deep structured populations of geographically isolated nipa (Nypa fruticans Wurmb.) in the Indo-West Pacific revealed using microsatellite markers
Source: Front Plant Sci. 2022 Oct 25;13:1038998. doi: 10.3389/fpls.2022.1038998 (PMC9641285; doi:10.3389/fpls.2022.1038998)
Supplement: Supplementary file 1 [file DataSheet_1.pdf]

Supplementary Materials:

Supplementary Material Table 1 (TABLE S1). Raw data of 445 samples from nipa populations in the Indo-West Pacific based on amplicon sizes using 18 SSR loci.

code	pop	Nfr11		Nfr14		Nfr17		Nfr22		Nfr25		Nfr26		Nfr27		Nfr30		Nfr31		Nfr39		Nfr40		Nfr55		Nfr56		Nfr58		Nfr59		Nfr60		Nfr69		Nfr72	
CD05	PHMS	292	292	268	266	300	300	206	206	268	268	224	224	292	292	234	234	104	104	276	270	162	162	192	192	174	168	294	284	162	162	234	234	264	264	184	184
CD07	PHMS	292	292	268	266	300	300	204	204	266	266	224	224	292	292	234	234	104	100	276	270	162	162	192	192	174	168	286	286	162	162	236	234	264	264	182	182
CD19	PHMS	292	286	270	270	312	300	204	204	268	268	224	224	292	292	238	238	100	100	280	272	162	162	192	192	176	176	294	282	180	162	238	238	270	270	186	186
TD04	PHMS	288	288	268	268	312	312	202	202	266	266	224	224	292	292	230	230	104	100	276	276	162	162	174	174	174	174	296	284	180	164	236	236	274	266	188	188
TD11	PHMS	292	288	268	268	312	300	204	204	284	264	262	262	292	292	224	224	104	100	278	274	164	160	192	192	166	166	284	284	178	162	238	238	264	264	186	186
TD15	PHMS	292	292	268	268	312	300	210	204	284	264	280	262	290	290	224	224	104	104	278	278	160	160	192	176	172	172	290	284	178	162	238	236	272	264	184	184
TD16	PHMS	292	292	268	268	312	300	204	204	264	264	280	262	292	292	226	226	104	100	278	274	160	160	192	192	172	172	292	284	178	162	238	238	264	264	186	186
TD20	PHMS	292	292	268	268	300	300	210	204	278	268	280	262	290	290	226	226	104	104	278	278	158	158	192	192	172	172	292	284	162	162	238	238	264	264	184	184
TD21	PHMS	292	292	268	268	312	300	210	204	278	264	280	262	292	292	224	224	104	100	278	278	164	164	190	190	172	172	292	284	180	180	236	236	264	264	186	186
TD22	PHMS	284	284	270	270	300	300	202	202	268	268	226	226	294	294	232	232	104	100	278	270	162	162	192	192	176	168	286	286	164	164	236	236	266	266	186	186
TD26	PHMS	290	290	268	268	300	300	204	204	284	268	224	224	292	292	230	230	100	100	278	278	162	162	192	192	176	176	288	284	178	178	240	236	268	268	186	186
TD27	PHMS	292	292	270	270	300	300	204	204	268	268	224	224	294	294	234	234	104	104	272	272	162	162	192	192	176	176	294	282	162	162	238	238	268	268	186	186
TD30	PHMS	290	290	270	270	300	300	204	204	298	284	224	224	292	292	234	234	104	100	278	278	162	162	192	174	176	170	294	284	180	162	240	236	270	270	188	188
TD31	PHMS	288	288	268	268	300	300	202	202	298	284	224	224	294	294	230	230	104	104	274	274	162	162	192	192	174	174	294	286	180	164	238	238	264	264	186	186
TD33	PHMS	288	288	268	268	310	310	204	204	264	264	228	228	292	292	234	234	104	104	280	274	162	162	192	192	166	166	294	282	162	162	236	236	278	268	184	184
BS03	PHME	292	288	268	268	312	312	204	204	266	266	226	226	292	292	230	230	104	104	276	276	162	162	192	192	168	168	292	284	162	162	234	234	272	272	186	186
BS04	PHME	290	290	266	266	312	312	204	204	262	262	224	224	292	292	230	230	104	100	276	276	162	162	192	180	174	168	294	284	162	162	236	236	272	264	186	186
BS05	PHME	288	288	266	266	300	300	202	202	0	0	226	226	294	294	230	230	106	106	278	278	162	162	194	194	166	166	290	284	162	162	240	240	274	268	186	186
BS06	PHME	292	292	270	270	312	300	204	204	268	268	226	226	292	282	232	232	104	100	276	258	160	160	192	192	168	168	290	284	178	164	236	236	274	266	186	186
BS07	PHME	290	286	268	268	312	312	206	206	266	266	226	226	298	298	230	230	104	104	276	276	162	162	194	194	168	168	292	284	162	162	234	234	264	264	186	186
BS08	PHME	288	288	268	268	312	300	204	204	268	268	224	224	294	294	230	230	104	104	278	278	158	158	194	194	170	170	294	282	162	162	238	238	280	266	186	186
BS09	PHME	294	294	0	0	302	302	204	204	268	268	226	226	292	292	236	236	104	102	276	258	160	160	190	190	174	162	284	284	164	164	0	0	274	266	184	184
BS10	PHME	0	0	0	0	310	300	204	204	268	268	228	228	292	292	232	232	104	102	276	260	160	160	190	190	162	162	294	294	164	164	236	232	272	266	184	184
BS11	PHME	292	292	268	268	310	302	204	204	268	268	226	226	292	282	236	230	104	104	276	258	160	160	192	192	174	172	294	282	164	164	236	236	272	266	182	182
BS15	PHME	288	288	268	268	310	310	204	204	266	266	226	226	292	282	236	230	106	106	274	274	166	156	192	180	166	166	294	282	162	162	238	238	278	278	186	186
BS16	PHME	284	284	0	0	312	302	204	204	268	268	228	228	292	282	238	232	106	106	278	278	162	162	194	194	166	166	284	284	164	164	240	240	278	270	186	186
BS19	PHME	288	284	266	266	310	310	202	202	268	264	224	224	292	282	238	232	104	104	278	278	162	162	192	192	166	166	294	282	162	162	238	238	274	266	186	186
BS20	PHME	292	288	270	270	312	312	204	204	0	0	226	226	292	282	236	230	104	104	274	274	0	0	192	192	166	166	294	282	162	162	240	240	276	268	184	184
BS21	PHME	288	288	268	268	312	312	202	202	266	266	224	224	292	282	236	230	104	104	278	278	158	158	192	180	164	164	294	284	162	162	240	240	276	276	188	188
BS22	PHME	290	290	272	272	310	300	206	206	268	266	226	226	282	282	0	0	106	106	276	276	162	162	192	192	174	174	284	284	164	164	240	240	278	268	186	186
BS23	PHME	284	284	266	266	312	300	202	202	266	266	224	224	292	292	238	232	104	104	278	278	162	162	194	194	166	166	290	284	162	162	238	238	274	266	186	186
BS24	PHME	294	290	0	0	306	306	206	206	286	268	226	226	298	298	236	236	106	102	278	258	164	164	194	194	0	0	290	284	180	164	236	230	268	268	186	186
BS26	PHME	294	294	266	266	314	302	204	204	268	268	224	224	292	282	240	236	106	106	278	278	162	162	192	192	172	168	286	286	164	164	242	242	274	266	188	188
PS09	PHMN1	288	284	268	268	294	294	204	204	278	266	224	224	290	290	236	236	104	100	276	272	162	162	190	190	168	168	294	282	164	164	236	236	266	266	186	186
PS18	PHMN1	288	288	268	266	294	294	204	204	278	268	224	224	292	292	236	236	104	104	276	276	162	162	190	190	174	174	294	284	180	164	236	236	266	266	186	186
PS23	PHMN1	288	288	268	266	310	310	204	204	266	266	226	226	292	292	236	236	104	104	276	272	162	162	190	190	168	168	288	282	180	164	236	236	266	266	186	186
PS25	PHMN1	288	284	268	268	314	302	202	202	268	268	224	224	292	282	236	230	104	104	278	278	162	162	192	192	168	168	294	284	180	162	240	240	276	266	186	186
PS28	PHMN1	288	288	268	264	314	302	202	202	278	266	224	224	292	282	236	236	104	100	276	276	162	162	192	192	168	168	294	284	162	162	240	240	266	266	188	188
PS33	PHMN1	292	292	268	268	300	300	204	204	268	268	224	224	292	292	238	238	104	104	276	276	162	162	192	192	168	168	294	282	178	162	234	228	272	264	184	184
CA04	PHMN2	292	288	268	268	312	300	204	204	266	266	226	226	292	292	236	236	104	100	278	278	162	162	194	194	176	170	294	286	162	162	238	238	264	264	184	184
CA17	PHMN2	288	284	268	266	314	302	202	202	266	266	224	224	290	290	236	236	104	100	278	278	162	162	192	192	174	174	294	284	180	176	240	240	276	276	186	186
CA18	PHMN2	288	288	268	266	312	312	204	204	284	268	226	226	292	292	230	226	104	100	274	274	162	162	194	194	172	172	294	284	162	162	236	236	278	270	188	188
CA20	PHMN2	290	290	268	268	312	312	204	204	266	266	228	228	292	282	230	226	104	100	276	276	162	162	194	194	176	172	294	282	180	180	238	230	278	270	188	188
CA23	PHMN2	288	284	268	266	312	312	202	202	278	278	226	226	292	292	236	236	104	100	278	278	162	162	192	192	168	168	292	286	180	164	240	234	276	276	188	188
CA28	PHMN2	288	284	268	268	312	300	204	204	268	266	224	224	282	282	236	236	100	100	278	278	162	162	190	190	176	176	288	284	164	164	238	234	268	268	188	188
MA06	PHMN2	288	288	268	266	310	310	204	204	268	268	226	226	292	282	230	230	104	104	278	278	162	162	190	190	168	168	292	282	180	164	234	234	274	266	188	188
MA08	PHMN2	288	284	268	268	312	300	204	204	268	268	224	224	292	282	230	230	104	104	278	278	162	162	190	190	174	168	292	282	164	164	238	238	276	268	188	188
MA16	PHMN2	288	288	268	266	312	312	204	204	266	266	228	228	290	290	230	230	104	104	276	276	162	162	194	194	176	172	294	282	180	164	240	232	278	270	190	190
MA18	PHMN2	288	288	268	266	312	312	202	202	264	264	224	224	290	290	230	230	100	100	276	276	162	162	192	192	174	168	294	284	162	162	240	240	266	266	188	188
[illegible]	[illegible]	[illegible]	[illegible]	[illegible]	[illegible]	[illegible]	[illegible]	[illegible]	[illegible]	[illegible]	[illegible]	[illegible]	[illegible]	[illegible]	[illegible]	[illegible]	[illegible]	[illegible]	[illegible]	[illegible]	[illegible]	[illegible]	[illegible]	[illegible]	[illegible]	[illegible]	[illegible]	[illegible]	[illegible]	[illegible]	[illegible]	[illegible]	[illegible]	[illegible]	[illegible]	[illegible]	[illegible]

Supplementary Material Table 2 (TABLE S2). Selection indices of microsatellite loci of nipa in the Indo-West Pacific.

LOCUS	SELECTION INDICES^z					Mode of Selection
	Probability	Log_{10} PO	Q-value	Alpha	F_{ST}	
Nfr11	0.0196039	-1.69906	0.695439	-0.0013309	0.33546	balancing
Nfr14	0.049810	-1.2805	0.32870	0.012079	0.33809	neutral
Nfr17	0.31126	-0.34493	0.20440	0.13109	0.36203	neutral
Nfr22	0.025005	-1.5910	0.64801	0.0025160	0.33623	neutral
Nfr25	0.014803	-1.8232	0.74640	-0.00029066	0.33566	balancing
Nfr26	0.017403	-1.7517	0.71458	-0.00022916	0.33567	balancing
Nfr27	0.038208	-1.4009	0.41914	0.0079080	0.33726	neutral
Nfr30	0.014003	-1.8477	0.75971	0.00075996	0.33585	neutral
Nfr31	0.036407	-1.4227	0.48720	-0.0078464	0.33432	balancing
Nfr39	0.020404	-1.6813	0.67352	-0.0011982	0.33549	balancing
Nfr40	0.029606	-1.5156	0.61829	0.0044628	0.33660	neutral
Nfr55	0.67233	0.31215	0.083317	-0.46395	0.25742	balancing
Nfr56	0.015203	-1.8114	0.73147	0.0011613	0.33593	neutral
Nfr58	0.99460	2.2652	0.0018670	-0.60893	0.23093	balancing
Nfr59	0.033007	-1.4668	0.58308	-0.0062288	0.33458	balancing
Nfr60	0.99980	3.6988	0.00010002	-0.76001	0.20837	balancing
Nfr69	1.0000	1000.0	0.0000	-0.91612	0.18678	balancing
Nfr72	0.033807	-1.4561	0.54042	-0.0054518	0.33472	balancing

^{z}PO = posterior odds; Alpha ≥0 and Q-value ≤0.05 (diversifying); Alpha ≥0 and Q-value>0.05 (neutral); Alpha <0 and Q-value>0.05 (balancing); Highlighted rows are significant at Q-value<0.05.

Supplementary Material Table 3 (TABLE S3). The multilocus genotype (MLG) diversity of nipa populations in Indo-West Pacific[z].

Pop	N	MLG	eMLG	SE	H	G	lambda	E.5	Hexp	Ia	p.Ia	rbarD	p.rD
PHMS	15	15	10.00	2.77E-07	2.708	15.00	0.933	1.000	0.637	0.755	0.003996	0.0454	0.003996
PHME	18	18	10.00	5.43E-07	2.890	18.00	0.944	1.000	0.700	0.519	0.000999	0.0313	0.000999
PHMN1	6	6	6.00	0.00E+00	1.792	6.00	0.833	1.000	0.513	0.583	0.082917	0.0381	0.082917
PHMN2	12	12	10.00	0.00E+00	2.485	12.00	0.917	1.000	0.602	0.750	0.000999	0.0483	0.000999
PHCE	19	19	10.00	2.51E-07	2.944	19.00	0.947	1.000	0.674	0.860	0.000999	0.0519	0.000999
PHLE	15	15	10.00	2.77E-07	2.708	15.00	0.933	1.000	0.659	0.523	0.002997	0.0316	0.002997
PHLW	18	18	10.00	5.43E-07	2.890	18.00	0.944	1.000	0.552	0.550	0.000999	0.0334	0.000999
PHPW	6	6	6.00	0.00E+00	1.792	6.00	0.833	1.000	0.655	3.728	0.000999	0.2276	0.000999
PHPE	9	9	9.00	0.00E+00	2.197	9.00	0.889	1.000	0.663	0.425	0.062937	0.0278	0.062937
MLVN1	15	15	10.00	2.77E-07	2.708	15.00	0.933	1.000	0.504	0.972	0.000999	0.0680	0.000999
MLVN2	21	21	10.00	0.00E+00	3.045	21.00	0.952	1.000	0.534	0.644	0.001998	0.0391	0.001998
MLTH1	15	15	10.00	2.77E-07	2.708	15.00	0.933	1.000	0.470	1.035	0.000999	0.0671	0.000999
MLTH2	16	15	9.62	4.84E-01	2.686	14.22	0.930	0.967	0.529	2.126	0.000999	0.1265	0.000999
MLTH3	16	16	10.00	0.00E+00	2.773	16.00	0.938	1.000	0.511	1.515	0.000999	0.0908	0.000999
MPNC1	20	19	9.76	4.25E-01	2.926	18.18	0.945	0.973	0.471	1.017	0.000999	0.0711	0.000999
MPNC2	15	15	10.00	2.77E-07	2.708	15.00	0.933	1.000	0.552	1.824	0.000999	0.1116	0.000999
MPNC3	16	16	10.00	0.00E+00	2.773	16.00	0.938	1.000	0.634	0.784	0.000999	0.0471	0.000999
MPEC1	26	26	10.00	1.09E-06	3.258	26.00	0.962	1.000	0.558	0.820	0.000999	0.0497	0.000999
MPEC2	15	14	9.57	4.95E-01	2.616	13.24	0.924	0.965	0.485	2.663	0.000999	0.2017	0.000999
MPEC3	15	15	10.00	2.77E-07	2.708	15.00	0.933	1.000	0.555	0.849	0.000999	0.0546	0.000999
MPEC4	15	15	10.00	2.77E-07	2.708	15.00	0.933	1.000	0.611	0.580	0.000999	0.0350	0.000999
MPEC5	15	15	10.00	2.77E-07	2.708	15.00	0.933	1.000	0.530	2.574	0.000999	0.1657	0.000999
MPSC	20	19	9.76	4.25E-01	2.926	18.18	0.945	0.973	0.556	0.940	0.000999	0.0574	0.000999
MPSW	21	21	10.00	0.00E+00	3.045	21.00	0.952	1.000	0.603	1.232	0.000999	0.0744	0.000999
INNJ	7	7	7.00	0.00E+00	1.946	7.00	0.857	1.000	0.664	2.393	0.000999	0.1435	0.000999
SLSW1	12	12	10.00	0.00E+00	2.485	12.00	0.917	1.000	0.426	2.148	0.000999	0.1441	0.000999
SLSW2	12	11	9.32	4.66E-01	2.369	10.29	0.903	0.958	0.388	1.879	0.000999	0.1345	0.000999
SLSW3	8	7	7.00	0.00E+00	1.906	6.40	0.844	0.943	0.293	0.923	0.004995	0.1028	0.004995
SLSW4	8	8	8.00	0.00E+00	2.079	8.00	0.875	1.000	0.430	1.241	0.000999	0.0897	0.000999
SLWC1	6	6	6.00	0.00E+00	1.792	6.00	0.833	1.000	0.341	0.397	0.197802	0.0284	0.197802
SLWC2	11	6	5.55	4.98E-01	1.421	2.95	0.661	0.622	0.204	5.109	0.000999	0.5731	0.000999
SLWC3	2	2	2.00	0.00E+00	0.693	2.00	0.500	1.000	0.370	NA	NA	NA	NA
**Total**	**445**	**431**	**9.99**	**1.16E-01**	**6.043**	**393.69**	**0.997**	**0.935**	**0.709**	**0.677**	**0.000999**	**0.0407**	**0.000999**

[z]Pop = population
N = census population size
MLG = number of unique multilocus genotype (MLG) observed
eMLG = number of expected MLG based on rarefaction at smallest N ≥ 10
SE = standard error of rarefaction analysis
H = Shannon-Wiener Index of MLG diversity
G = Stoddart and Taylor's Index of MLG diversity
lambda = Simpson's Index
E.5 = Evenness
Hexp = Nei's (1978) expected heterozygosity
Ia = Index of association
p.Ia = p-value for Ia
rbarD = standardized index of association
p,rD = p-value for rbarD
NA = values for SL_WC3 could not be plotted

Supplementary Material Table 4 (TABLE S4). Statistical analysis results of Mantel correlogram.

Distance class (W)	Class index	Number of distance classes	Mantel correlation	Pr (Mantel)	Pr (corrected)	Stat. signif.
1	1.29E+00	2.47E+04	3.00E-01	0.001	0.000999	***
2	3.87E+00	1.68E+04	9.17E-02	0.001	0.001998	**
3	6.45E+00	2.48E+04	9.98E-02	0.001	0.002997	**
4	9.04E+00	1.32E+04	7.83E-02	0.001	0.003996	**
5	1.16E+01	8.54E+03	-1.06E-02	0.160	0.159840	ns
6	1.42E+01	2.46E+03	2.24E-02	0.006	0.011988	*
7	1.68E+01	5.87E+03	2.26E-02	0.021	0.041958	*
8	1.94E+01	1.69E+04	-6.19E-02	0.001	0.007992	**
9	2.19E+01	3.85E+04	-1.11E-01	0.001	0.008991	**
10	2.45E+01	2.24E+04	-8.58E-02	0.001	0.009990	**
11	2.71E+01	8.74E+03	-1.37E-01	0.001	0.010989	*
12	2.97E+01	8.26E+02	-4.38E-02	0.001	0.011988	*
13	3.23E+01	0.00E+00	NA	NA	NA	-
14	3.49E+01	0.00E+00	NA	NA	NA	-
15	3.74E+01	6.96E+02	-5.51E-02	0.001	0.012987	*
16	4.00E+01	2.77E+03	-1.20E-01	0.001	0.013986	*
17	4.26E+01	3.12E+03	-1.15E-01	0.001	NA	-
18	4.52E+01	7.34E+03	-1.58E-01	0.001	NA	-

Signif. codes: *** 0.001 ** 0.01 *0.05 ^{ns}not significant

Supplementary Material Table 5 (TABLE S5). The list of first-generation (F_0) immigrants detected among nipa populations in the Indo-West Pacific based on multilocus genotypes[a].

SAMPLE	HOME POPULATION	-log (L_home/ L_max)	Probability	-log(L)	REFERENCE POPULATION
TD04	PHMS	8.185	0.001	19.089	PHMN2
TD22	PHMS	6.866	0.002	23.492	PHME
TD33	PHMS	6.538	0.001	19.090	PHCE
BS09	PHME	2.713	0.003	18.074	MPNC3
BS21	PHME	3.694	0.003	21.105	PHMN2
PS33	PHMN1	12.477	0.001	18.002	PHMS
CA04	PHMN2	3.308	0.008	18.727	PHCE
CA17	PHMN2	8.567	0.000	17.102	PHMN1
BB07	PHCE	3.193	0.004	26.148	PHMS
CB04	PHCE	3.508	0.004	19.171	PHPE
CB19	PHCE	2.878	0.005	18.898	PHPE
CB34	PHCE	2.009	0.010	20.415	INNJ
LQ18	PHLE	3.344	0.000	26.391	MLTH1
LQ21	PHLE	2.581	0.001	19.985	PHCE
NP03	PHPW	16.331	0.002	28.041	PHMS
MP08	PHPE	9.570	0.001	18.909	MPEC5
SP04	PHPE	5.457	0.004	23.993	MLVN2
HC22	MLVN1	15.240	0.000	11.119	MLVN2
BT3-02	MLVN2	3.221	0.006	18.048	MLVN1
BT4-19	MLVN2	3.836	0.002	28.081	MPNC3
BT4-26	MLVN2	2.924	0.006	14.889	MLVN1
TH1-03	MLTH1	10.503	0.000	14.246	MLTH3
TH1-09	MLTH1	7.212	0.003	15.313	MLTH3
TH2-10	MLTH1	5.685	0.005	12.609	MLTH2
TH3-05	MLTH2	16.330	0.000	27.497	INNJ
TH15-01	MLTH2	8.598	0.002	20.215	MLTH1
TH16-03	MLTH2	6.501	0.005	14.043	INNJ
TH4-06	MLTH3	13.109	0.001	20.016	INNJ
TH4-09	MLTH3	7.548	0.002	11.525	MLTH2
TH4-19	MLTH3	13.666	0.000	18.600	MPNC3
TH5-05	MPNC1	1.230	0.005	14.315	MLTH1
TH10-01	MPNC2	2.526	0.007	18.347	MPEC3
TH11-04	MPNC2	2.830	0.010	11.433	MPNC3
TH11-07	MPNC2	4.572	0.002	16.797	MPNC3
TH14-09	MPNC3	5.348	0.004	20.815	SLSW4
TH14-11	MPNC3	7.772	0.001	18.693	INNJ

[a] -log(L-home/L_max) (likelihood ratio); probability, significant at $p<0.01$; -log(L) (genetic distance); reference population (population of origin) with population codes found in Table 2.

Supplementary Material Table 5 (TABLE S5). (cont.)[a]

SAMPLE	HOME POPULATION	-log (L_home/ L_max)	Probability	-log(L)	REFERENCE POPULATION
TH14-13	MPNC3	5.850	0.002	21.958	MPSW
TH14-20	MPNC3	4.517	0.004	17.020	MPEC1
MA8-09	MPEC1	6.348	0.000	13.879	MLTH3
MA8-16	MPEC1	7.644	0.000	20.625	MPNC3
MA8-19	MPEC1	3.054	0.006	16.067	MPEC3
MA9-20	MPEC1	3.506	0.005	21.504	MLTH2
MA5-16	MPEC3	5.327	0.001	19.186	MPEC5
MA5-17	MPEC3	4.714	0.001	14.219	MPEC1
MA5-25	MPEC3	5.381	0.001	19.159	PHPE
MA3-16	MPEC4	3.374	0.004	22.747	MLVN1
MA3-26	MPEC4	1.701	0.009	17.752	MPEC5
MA4-04	MPEC5	6.617	0.001	18.751	MPEC4
SG19	MPSC	0.471	0.001	17.434	MPSW
MA10-19	MPSW	4.377	0.000	25.640	INNJ
MA10-22	MPSW	3.479	0.000	16.895	MPSC
IN1-02	INNJ	4.669	0.005	32.741	PHME
SL1-10	SLSW1	5.486	0.007	13.657	SLSW2
SL1-12	SLSW1	6.660	0.004	11.973	SLSW4
SL5-01	SLSW4	8.547	0.005	17.084	SLWC1
SL6-03	SLWC1	15.988	0.000	12.654	SLSW2
SL6-08	SLWC1	12.050	0.003	3.730	SLWC2
SL7-04	SLWC2	8.198	0.003	6.021	SLWC3
SL10-01	SLWC3	37.608	0.000	2.392	SLWC2
SL10-02	SLWC3	25.880	0.000	14.120	SLSW1

[a] -log(L-home/L_max) (likelihood ratio); probability, significant at $p<0.01$; -log(L) (genetic distance); reference population (population of origin) with population codes found in Table 2.
